# Severe Abdominal Pain due to Cough‐Induced Internal Oblique Muscle Hematoma: A Case Report

**DOI:** 10.1002/ccr3.70910

**Published:** 2025-09-22

**Authors:** Oguz Kagan Sahin, Ayse Gulsah Kaya, Eshita Sharma

**Affiliations:** ^1^ Edremit State Hospital Balikesir Turkey; ^2^ University of South Florida Tampa Florida USA; ^3^ David Geffen School of Medicine at UCLA Los Angeles California USA

**Keywords:** acute abdominal pain, conservative management, cough‐induced injury, CT imaging, spontaneous muscle hematoma

## Abstract

Spontaneous internal oblique muscle hematomas are rare and may closely mimic acute abdominal emergencies. This case emphasizes the importance of including them in the differential diagnosis of acute abdominal pain. Clinicians should consider this diagnosis in patients with cough‐related abdominal pain to prevent misdiagnosis and unnecessary surgical interventions.

## 1 Introduction

Hematomas of the abdominal wall musculature, while uncommon, pose a diagnostic challenge in patients experiencing acute abdominal pain. Although the rectus sheath is the most commonly affected area, accounting for most abdominal wall hematomas, hematomas involving the lateral abdominal wall, such as the internal oblique muscle, are considerably rarer [1, 2]. These hematomas can mimic other causes of an acute abdomen and are often associated with identifiable risk factors, including anticoagulation therapy, trauma, or increased intra‐abdominal pressure due to activities like coughing, straining, or vomiting [1, 2, 3]. A precise and timely diagnosis is essential to avoid unnecessary surgical intervention and optimize patient care.

This case report highlights a unique instance of a large spontaneous hematoma in the left internal oblique muscle, which is less commonly affected than the rectus sheath, in a patient with a history of chronic cough. This emphasizes the importance of considering rare causes of abdominal pain, even when there is no apparent trauma or standard risk factors.

## 2 Case Presentation

### 2.1 Case History/Examination

A 47‐year‐old male presented to the Emergency Department with the acute onset of sharp left‐sided abdominal pain, which had started earlier that day. The patient denied any history of direct abdominal trauma, recent surgical procedures, anticoagulant use, or intramuscular injections. His medical history was notable for chronic hypertension and a previous episode of urolithiasis 2 years prior. He characterized the current pain as similar to the discomfort experienced during his previous urolithiasis episode. Furthermore, he reported a three‐day history of severe coughing, a sore throat, and generalized fatigue.

Upon physical examination, the patient was afebrile with a temperature of 36.8°C. His vital signs were stable, with a blood pressure of 130/78 mmHg, a pulse rate of 84 beats per minute, and a respiratory rate of 18 breaths per minute. The patient's skin appeared normal in color, and the abdominal examination revealed diffuse left‐sided lateral abdominal tenderness without costovertebral angle tenderness.

### 2.2 Differential Diagnosis, Investigations, and Treatment

Laboratory tests and imaging studies were ordered, and initial pain management included intravenous administration of paracetamol, dexketoprofen, and tramadol administered at varying intervals. Despite these interventions, the patient's reported pain persisted, albeit at a lower intensity than before analgesic administration. The laboratory findings revealed the following: hematocrit at 41%, hemoglobin at 13.7 g/dL, platelet count at 256,000/mm^3^, white blood cell count (WBC) at 13,250/mm^3^, activated partial thromboplastin time (aPTT) of 33 s, prothrombin time (PT) of 26 s, and an international normalized ratio (INR) of 1.2. These values indicated no coagulopathy or significant anemia, supporting the diagnosis of a localized hematoma rather than a systemic bleeding disorder.

**FIGURE 1:**
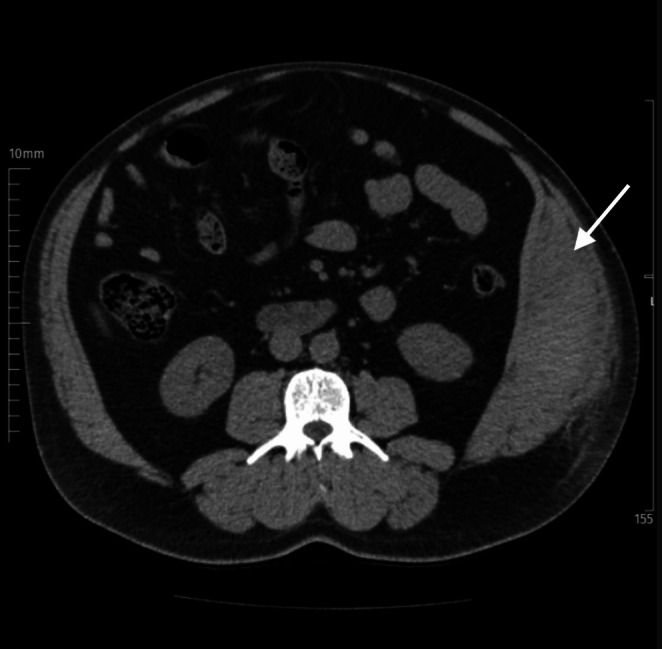
Computed tomography of the abdomen showing a 10.5 × 6.5 × 5.5 cm hematoma in the left internal oblique muscle (arrow).

Given the persistence of the pain, an initial contrast‐enhanced abdominal CT scan was deemed necessary. However, a non‐contrast CT scan was subsequently performed since the patient refused contrast material. The findings revealed a hyperdense mass within the left internal oblique muscle, measuring 10.5 × 6.5 × 5.5 cm (see Figure 1). The large size and well‐defined margins were consistent with a hematoma rather than a neoplastic lesion or inflammatory pathology, such as an abscess or infection. These findings suggest an oblique muscle hematoma, likely due to excessive coughing and resultant muscle strain.

### 2.3 Conclusion and Results (Outcome and Follow‐Up)

After consulting with the general surgery and orthopedics teams, the patient was subsequently admitted to the general surgery inpatient service for observation to rule out potential complications, such as hematoma expansion, and to continuously monitor hemodynamic stability. Given the patient's stability and lack of hematoma progression, conservative management was initiated, including bed rest, analgesics, and intravenous fluids. Daily hemogram follow‐ups indicated no significant decline, and the patient was discharged after 2 days in stable condition with instructions for follow‐up in the outpatient clinic.

**FIGURE 2:**
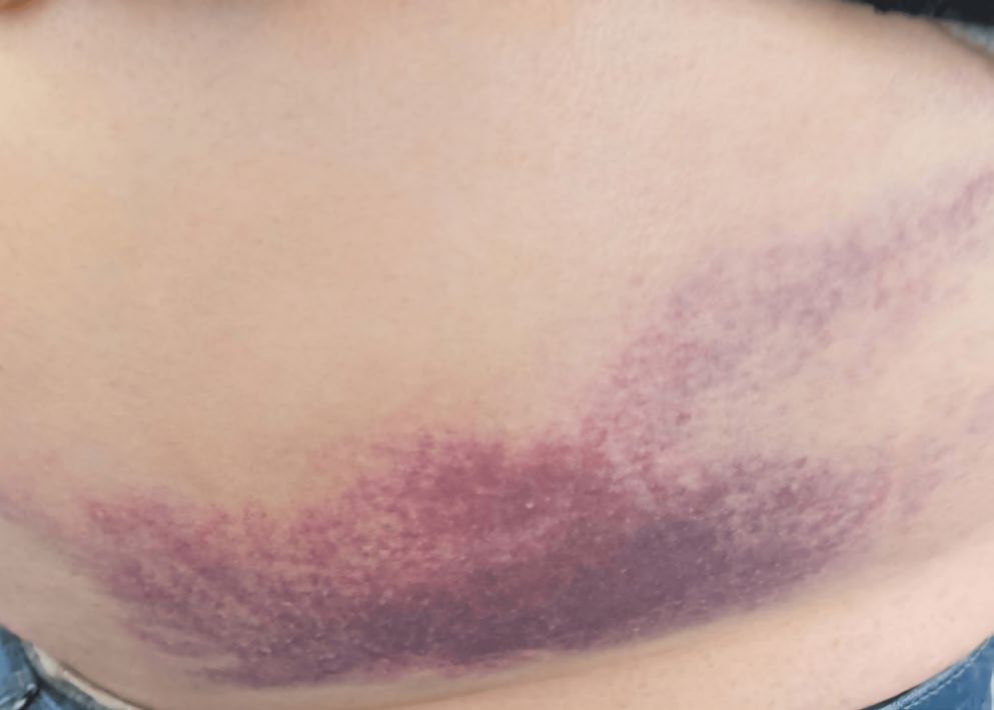
Patient's ecchymosis on the left lateral side of the abdomen.

**FIGURE 3:**
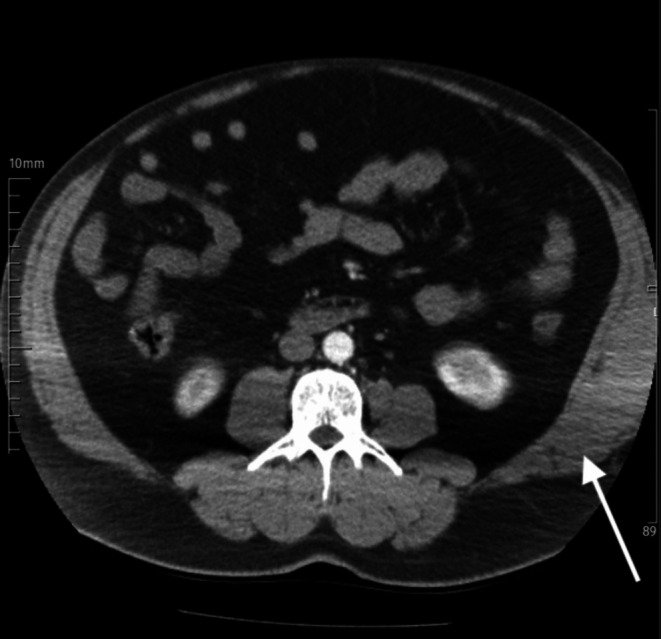
Follow‐up computed tomography performed 2 weeks later showing that the size of the hematoma decreased (arrow).

Two days after his discharge, the patient revisited the emergency department due to his concerns over a color change in the affected area. During the evaluation, the patient reported improved abdominal pain; however, a large ecchymosis was observed on the patient's left lateral abdominal skin (Figure 2). After 2 weeks, a follow‐up CT scan showed a reduction in the size of the hematoma (Figure 3).

## 3 Discussion

Abdominal wall hematomas are an uncommon cause of acute abdominal pain. This case illustrates several diagnostic pitfalls, as the lateralized abdominal pain could be easily mistaken for more common intra‐abdominal pathologies such as appendicitis, diverticulitis, torsion, tumors, or hernias [4, 5]. Rectus sheath hematomas are the most commonly encountered form, whereas internal oblique muscle hematomas are rare [3, 5]. Risk factors include anticoagulation therapy, bleeding disorders, pregnancy, intense physical activity, obesity, and prior surgeries [1, 2, 3].

Coughing is a natural reflex; however, prolonged forceful coughing can lead to a sudden increase in intra‐abdominal pressure, rupturing small intramuscular vessels and causing hematoma formation [6]. The pathophysiological mechanism involves increased intra‐abdominal pressure during vigorous coughing, rupturing small muscular vessels, particularly in high‐contractility muscles like the internal oblique.

The present case describes a spontaneous internal oblique muscle hematoma in a middle‐aged male, likely precipitated by repeated episodes of intense coughing. The literature reports several similar cases of cough‐induced internal oblique muscle hematomas [3, 5, 7, 8, 9, 10, 11, 12, 13].

Clinically, upon initial examination, the patient presented with lateral abdominal pain without any visible signs of bruising. This observation aligns with previous reports in which ecchymosis tends to develop later in the course of the hematoma; in a case series, 17% of the patients with abdominal wall hematoma present with an abdominal wall ecchymosis [2, 12]. The patient's persistent pain, despite multiple analgesic administrations, was a key feature in our case, which prompted further imaging.

The role of imaging in diagnosing abdominal wall hematomas is critical. Both ultrasound and CT imaging are viable diagnostic tools; however, CT provides superior accuracy in detecting the location and spread of the hematoma [14]. Initial non‐contrast CT scans typically reveal a hyperdense mass consistent with hematoma, while contrast‐enhanced CT helps differentiate active bleeding from a stable hematoma [14, 15]. In our case, the patient was hemodynamically stable with no significant decline in hemoglobin levels, supporting the decision for conservative management. This is consistent with existing literature advocating conservative treatment in stable patients without hemodynamic compromise [16].

Management strategies include bed rest, analgesia, and serial monitoring for resolution or expansion. The management decision‐making process is largely guided by the hematoma's severity, which can be classified based on CT findings (Table 1). Grade I and II hematomas are typically managed conservatively with bed rest and analgesia. In contrast, Grade III hematomas may require more intensive monitoring, potential embolization, or surgical intervention in cases of ongoing hemorrhage [16]. Surgical intervention or transcatheter arterial embolization is generally reserved for cases with ongoing hemorrhage, signs of peritoneal irritation, hemodynamic instability, or when conservative treatment options fail [14, 16]. In our case, the stable hemodynamic condition and subsequent symptom improvement allowed for a conservative management approach with outpatient follow‐up. This highlights the importance of a stepwise, conservative approach for hemodynamically stable patients while reserving surgical options for more severe cases.

**TABLE 1:** Summary of CT‐based grading of hematomas.

Grade	CT findings/description	Typical management/hospitalization
I	Small, contained within the muscle	Conservative management (outpatient bed rest, analgesia).
II	Larger, extending between muscle layers	Conservative management (outpatient bed rest, analgesia). Hospitalization for monitoring if risk factors are present.
III	Extensive, with possible involvement of adjacent structures	Hospitalization for intensive monitoring. May require interventional radiology (embolization) or surgery for ongoing hemorrhage, hemodynamic instability, or peritoneal irritation.

This case is notable for two key features: the unusual location of the hematoma within the internal oblique muscle, unlike the typical rectus sheath presentation, and the delayed appearance of ecchymosis, which underscores the evolving nature of abdominal wall hematomas and the need for follow‐up. It also shows the importance of considering musculoskeletal sources of abdominal pain in patients with recent cough episodes, particularly when they present with lateralized pain resistant to standard analgesia.

In conclusion, internal oblique muscle hematomas must be included in the differential diagnosis of acute abdominal pain in clinical practice, particularly in patients with risk factors such as intense coughing or underlying hypertension. Early imaging is essential for accurate diagnosis and to guide appropriate management. Conservative treatment remains the preferred approach in hemodynamically stable patients, with close monitoring to ensure resolution. Greater awareness of this condition can help prevent unnecessary surgical interventions and improve patient outcomes.

## Author Contributions

**Oguz Kagan Sahin:** conceptualization, data curation, investigation, methodology, project administration, resources, supervision, writing – original draft, writing – review and editing. **Ayse Gulsah Kaya:** conceptualization, data curation, methodology, project administration, supervision, writing – original draft, writing – review and editing. **Eshita Sharma:** conceptualization, data curation, methodology, project administration, resources, supervision, writing – review and editing.

## Ethics Statement

The authors have nothing to report.

## Consent

The patient provided written informed consent to publish this report, in line with the journal's patient consent policy.

## Conflicts of Interest

The authors declare no conflicts of interest.

## Data Availability

The data that support the findings of this study are available from the corresponding author upon reasonable request.
